# Prevalence of functional dependence and chronic diseases in the community-dwelling Brazilian older adults: an analysis by dependence severity and multimorbidity pattern

**DOI:** 10.1186/s12889-023-17564-w

**Published:** 2024-01-10

**Authors:** Wanderley Matos Reis Júnior, Luciano Nery Ferreira, Cynthia Goulart Molina-Bastos, José Patrício Bispo Júnior, Helca Franciolli Teixeira Reis, Bárbara Niegia Garcia Goulart

**Affiliations:** 1 Department of Health 1, State University of Southwest Bahia, Jequié, Bahia Brazil; 2 Department of Social Medicine, Federal University of Rio Grande Do Sul, Rio Grande Do Sul, Porto Alegre, Brazil; 3 Federal University of Rio Grande Do Sul, Rio Grande Do Sul. Rua Ramiro Barcelos, 2777 Room 307, Porto Alegre, RS CEP 90035-003 Brazil; 4 Federal University of Bahia, Multidisciplinary Health Institute, Vitória da Conquista, Brazil

**Keywords:** Prevalence studies, Chronic disease, Functional status, Older adults

## Abstract

**Background:**

Despite the advancements in knowledge about health care for older adults, essential gaps persist regarding the effects of chronic diseases as epidemiological markers of the state of functional dependence. This study aimed to identify the prevalence of moderate and severe functional dependence in Brazilian older adults and its association with chronic diseases and verify the multimorbidity patterns by dependence status.

**Methods:**

This cross-sectional analytical study used data from 11,177 community-dwelling Brazilian older adults from the 2013 National Health Survey conducted in Brazil. The dependent variables were moderate and severe functional dependence in basic activities of daily living (BADLs) and instrumental ADLs (IADLs). The independent variables were defined based on the questions applied to measure each morbidity in a self-reported manner and asked, "Has a doctor ever diagnosed you as having (each disease)? Multimorbidity was simultaneously considered present for older adults with ≥ 2 chronic morbidities. The association between functional dependence on BADLs and IADLs separately by severity and the independent variables was verified from crude and adjusted estimates of the point prevalence ratios and their 95% confidence intervals using the regression model Poisson with robust variance. To group diseases into patterns, exploratory factor analysis was used.

**Results:**

The prevalences of moderate and severe BADL dependence were 10.2% (95% CI, 9.6–10.7) and 4.8% (95% CI, 4.4–5.2), respectively. Moderate and severe IADL dependence prevalences were 13.8% (95% CI, 13.1–14.4) and 15.6% (95% CI, 14.9%–16.2), respectively. When changing the condition from moderate to severe dependence in BADLs, in the presence of other mental illnesses and stroke, the probability of dependence increased more than four times in the case of other mental illnesses and more than five times for stroke. There was a linear trend for dependence severity, both moderate and severe, whereas, for severe dependence on IADLs, this same factor maintained a linear trend toward an increase in probability as the number of diseases simultaneously increased.

**Conclusions:**

Chronic diseases are associated with functional dependence, with greater emphasis on mental illnesses and stroke in severe disability, considering their acute adverse effects.

## Background

World population aging is already observed in several countries worldwide and is a relevant and complex public health issue in developing countries [1, 2]. This phenomenon has several effects, such as an increase in the burden of chronic diseases and the prevalence of multimorbidity [3], whose effects on the health status of older adults are significantly more evident as the accumulation of chronic diseases over the lifetime has increased, both in prevalence and incidence [4, 5]. These demand more inputs, actions, and use of health services, implying a significant portion of health expenses [6].

Among the multiple challenges that older adults have to deal with, multimorbidity, which is characterized by the co-occurrence of two or more chronic diseases simultaneously, with its complexity and consequences, requires adequate and sustained management with short, medium, and long-term strategies, both for evaluation and monitoring and for various treatments in health and social support [4, 7].

The co-occurrence of these chronic diseases, associated with advancing biological age, can result in functional decline, whose burden increases as necessary interventions are not performed and investments in chronic disease prevention and timely health promotion are neglected [5, 8].

Approximately 70% of Brazilians have at least one chronic disease by the age of 60 years, indicating that the burden of several chronic diseases is high, growing, and worrying among the older adult population [9]. Chronic diseases are strongly related to direct healthcare expenditure and healthcare service use. As many chronic diseases the patient has, the more they use health services and the more expensive the treatment [10].

Thus, the development of chronic diseases may represent an initial change in functional independence, following a course that is often progressive with the development of dependence, whose trajectory goes through mild, moderate, and severe dependence, evolving to total disability [11–13].

Usually, a binary classification to refer to functional dependence, such as dependent and non-dependent, has been valid, for example, in estimating the prevalence of disability and directing specific interventions among dependents. However, this approach does not consider the heterogeneity associated with disability severity, which is often observed in older adults [14].

In general, current studies have focused on the association between each chronic disease in isolation and dependence on activities in older adults, considering functional dependence as an isolated and dichotomous variable. However, multimorbidity and functional dependence results still require further investigation to determine the synergy between its agents [6, 9].

Therefore, functional dependence, as an indirect marker of vitality or limitations, varies significantly in the way it is experienced by each older adult concerning the combination of chronic diseases they have, the time they live with these problems, and other variables related to lifestyle and social support. The functional assessment includes Basic activities of daily living (BADLs) and Instrumental activities of daily living (IADLs). Also, it seeks to assess at what stage diseases and injuries prevent older adults from performing their daily activities, independently and autonomously. To this end, the need for help or assistance from others or adaptations is considered, contributing to decision-making and planning of more coherent and accurate actions. BADL and IADL allow the construction of diagnoses, prognoses, and coherent clinical strategies, which guide the decision-making for specialized care [6, 9, 10, 13, 15, 16].

In addition, functional dependence has variations beyond the dichotomous assessment of having or not having functional dependence. In this context, this study aimed to identify the prevalence of functional dependence in Brazilian older adults and its association with chronic diseases and to verify the patterns of multimorbidity by the status of dependence, comparing independent, moderately dependent, and severely dependent individuals.

## Methods

### Study design

This was a cross-sectional analytical study, as recommended by the Strengthening the Reporting of Observational Studies in Epidemiology (STROBE) [17].

### Population and sample

This study used a subsample from the National Health Survey (NHS) conducted in Brazil in 2013 [18, 19]. Considering the purposes of this study, the sample was restricted to all community older adults, comprising 11,177 older adults, aged > 60 years, who responded to the applied questionnaires, and who were randomly selected, according to the methodology described previously [20].

### Study variables

The outcomes analyzed were moderate and severe functional dependence in basic activities of daily living (BADLs) and instrumental ADLs (IADLs), analyzed independently. Dependence on BADLs was identified using the Katz Index [21], which assesses partial or total help for several activities, such as eating, bathing, going to the bathroom, getting dressed, walking around the house alone, and getting in/out of bed. Dependence on IADLs was measured using the Lawton Scale [22] for several activities, such as shopping, taking care of finances, taking medications, going to the doctor alone, and using transportation. For BADLs, the older adults were classified as moderately dependent when they reached values ≥ 3 and ≤ 5 points, severely dependent when they reached values ≤ 2 points, and independent when they reached 6 points. For IADLs, older adults were classified as moderately dependent when they reached values ≥ 3 and < 5 points, severely dependent when they reached values ≤ 2 points, and independent when they reached values of 5 points [22, 23].

Exposure variables were defined based on the questions applied to measure each morbidity, which were self-reported based on a medical diagnosis. The older adults were asked the following question: “Have any doctor diagnosed you as having (each disease)?” The answer options for each question were “yes” or “no.” The following 14 chronic diseases included in the Brazilian NHS were considered: heart disease, asthma or asthmatic bronchitis, chronic obstructive pulmonary disease (COPD), systemic arterial hypertension (SAH), diabetes, hypercholesterolemia, stroke, cancer, chronic kidney failure, arthritis or rheumatism, chronic back problems, work-related musculoskeletal disorders (WMSDs), depression, and other mental illnesses, including schizophrenia, bipolar disorder, psychosis, and obsessive–compulsive disorder. The number of chronic diseases ranged from no chronic diseases to more than four. Multimorbidity derived from the categorization of the variable chronic diseases was considered present for those older adults with ≥ 2 chronic morbidities simultaneously [24, 25]. As adjustment variables, all chronic diseases were considered simultaneously in model 1. For model 2, the variables of model 1 were considered (*p* < 0.05), along with the number of chronic diseases and sociodemographic factors (sex, age, education, marital status, race/ skin color).

### Data analyses

All analyses were performed using weighted data that considered the sample characteristics. Descriptive analyses were performed to calculate the prevalence and respective 95% confidence intervals (95% CIs). The association between functional dependence in BADLs and IADLs by *status* and the exposure variables was verified from crude and adjusted estimates of the prevalence ratio by point and 95% CI using the model Poisson regression with robust variance.

The adjusted analysis included the variables that showed statistical significance in the crude analyses, with a “forward” selection strategy in determining the outcomes [26], with significance for *p* values ≤ 0.05. Statistical analyses were performed using the *SPSS* version 26.0.

To group diseases patterns, exploratory factor analysis was performed, stratified by dependence status [27, 28]. The exploratory factor analysis allowed the identification of coexisting diseases trends in a set of variables with causal factors that are potentially common and interpreted as multimorbidity patterns.

The tetrachoric correlation coefficient was used in the analysis because it is more appropriate than the Pearson correlation for dichotomous outcomes [29]. Sampling adequacy was assessed using the Kaiser–Meyer–Olkin (KMO) test, considered adequate if the index was ≥ 0.70, and the Bartlett spherical test, considered adequate if the *p*-value was ≤ 0.05 [28, 30]. A cattle graph (scree plot), which represented the eigenvalues of the correlation matrix in descending order, was used to establish the number of factors to be maintained.

The extracted factor number corresponds to the eigenvalue that produces the inflection point in the curve (eigenvalue > 1) and minimum explained variance (> 10% for each component). Variables were defined as associated with a factor if it had a factor loading ≥ 0.30 [28], as the closer to 1, the greater the association. Oblique rotation (Varimax) was performed to allow for a better interpretation of the factor analysis [28].

### Ethical considerations

This study was conducted in accordance with ethical research standards using data extracted from the NHS and approved by the National Research Ethics Committee in June 2013 (no. 328159). All individuals included in the final sample agreed to participate in this study and signed an informed consent form.

## Results

Of the 11,177 community-dwelling Brazilian older adults participants in this study, 6.622 (59.2%) were female. The mean age of the participants was 69.8 (standard deviation, 7.8) years. The prevalences of moderate and severe BADL dependence were 10.2% (95% CI, 9.6–10.7) and 4.8% (95% CI, 4.4–5.2), respectively. The prevalences were 13.8% (95% CI, 13.1–14.4) and 15.6% (95% CI, 14.9%–16.2) in moderate and severe IADL dependence, respectively.

Above 80 years of age, around 2.5% of the older adult participants in the study had moderate difficulty with BADL, while for those seriously dependent, this number corresponds to 1.9%. For IADL, impairment percentages for older people aged under 79 years accumulate percentages of 9.5% for severe impairment and above 11.0% for moderate impairment. For other characteristics, see Table 1.

**Table 1:** Prevalence of moderate and severe dependence in BADLs and IADLs according to sociodemographic characteristics in community-dwelling Brazilian older adults

Variables	**BADLs**				**IADLs**			
	Independent (*n* = 9.493)	Moderately dependent (*n* = 1.143)	Severely dependent (*n* = 541)	*P*-value^*^	Independent (*n* = 7.893)	Moderately dependent (*n* = 1.540)	Severely dependent (*n* = 1.744)	*P*-value^*^
	n (%)	n (%)	n (%)		n (%)	n (%)	n (%)	
**Sex**				0.001				< 0.001
Male	3.937 (35.3)	428 (3.8)	190 (1.7)		3.472 (31.1)	547 (4.9)	536 (4.8)	
Female	5.556 (49.7)	715 (6.4)	351 (3.1)		4.421 (39.6)	993 (8.9)	1.208 (10.8)	
**Age group**				< 0.001				< 0.001
60–69	5.623 (50.4)	486 (4.3)	129 (1.2)		5.148 (46.1)	618 (5.5)	472 (4.2)	
70–79	2.884 (25.8)	372 (3.4)	185 (1.7)		2.220 (19.9)	631 (5.6)	590 (5.3)	
80–89	886 (7.9)	236 (2.1)	171 (1.5)		494 (4.4)	261 (2.3)	538 (4.8)	
≥ 90	100 (0.9)	49 (0.4)	56 (0.4)		31.0 (0.3)	30 (0.3)	144 (1.3)	
**Education**				< 0.001				< 0.001
Higher	1.077 (12,5)	62 (0.7)	28 (0.3)		1.027 (11.9)	77 (0.9)	63 (0.7)	
Average	2.887 (33.5)	266 (3.1)	114 (1.3)		2.610 (30.2)	333 (3.9)	324 (3.8)	
Primary	3.535 (40.9)	473 (5.5)	198 (2.2)		2.871 (33.2)	652 (7.5)	683 (7.9)	
**Marital status**				< 0.001				< 0.001
With partner	4.442 (39.7)	436 (3.9)	170 (1.5)		3.934 (35.2)	569 (5.1)	545 (4.9)	
No partner	5.051 (45.2)	707 (6.3)	371 (3.3)		3.959 (35.4)	971 (8.7)	1.199 (10.7)	
**Race/color**				0.900				0.028
White	4.512 (40.4)	540 (4.8)	262 (2.3)		3.816 (34.1)	696 (6.2)	802 (7.2)	
Not White	4.981 (44.6)	603 (5.4)	279 (2.5)		4.077 (36.5)	844 (7.6)	942 (8.4)	

*BADLs* Basic activities of daily living, *IADLs* Instrumental activities of daily living

^*^Pearson’s chi-squared test – significance *p*-value < 0.005

In Table 2, the crude analysis for the association of chronic diseases and multimorbidity with moderate and severe dependence in BADL and IADL, it can be seen that for moderate dependence, all chronic diseases showed a significant association only in BADL, but not in IADL (see Hypercholesterolemia and WMSDs). For severe dependence on BADLs, the chronic diseases that showed the most significant association were stroke, with eight times the probability of severe dependence, and other mental illnesses, with more than three times the probability. Regarding the number of coexisting chronic diseases multimorbidity, there was a linear trend for dependence severity, both moderate and severe, in BADLs. The more chronic diseases that coexist, the greater the association with moderate and severe dependence in the older adults in the crude analysis.

**Table 2:** Crude analysis of the association between chronic diseases^d^ and moderate and severe dependence in community-dwelling Brazilian older adults

	Moderate dependence			Severe dependence		
**Exposure variables**	Not n (%)	Yes n (%)	PR_crude_ (95% CI)	Not n (%)	Yes n (%)	PR_crude_ (95% CI)
**BADLs**						
SAH (ref. no.)	439 (38.8)	691 (61.2)	1.60 (1.43–1.79)^a^	225 (41.9)	312 (58.1)	1.46 (1.23–1.72)^a^
Diabetes (ref. no.)	823 (75.4)	269 (24.6)	1.55 (1.36–1.76)^a^	368 (71.7)	145 (28.3)	1.90 (1.58–2.29)^a^
Hypercholesterolemia (ref. no.)	736 (68.7)	335 (31.3)	1.35 (1.20–1.53)^a^	394 (77.9)	112 (22.1)	0.88 (0.72–1.09)^c^
Heart diseases (ref. no.)	961 (84.1)	182 (15.9)	1.77 (1.53–2.04)^a^	446 (82.4)	95 (17.6)	2.06 (1.66–2.54)^a^
Stroke (ref. no.)	1,029 (90.0)	114 (10.0)	2.72 (2.31–3.22)^a^	393 (72.6)	148 (27.4)	8.03 (6.82–9.46)^a^
Bronchial asthma (ref. no.)	1,067 (93.4)	76 (6.6)	1.59 (1.28–1.97)^a^	501 (92.6)	40 (7.4)	1.83 (1.34–2.49)^a^
Rheumatoid arthritis (ref. no.)	798 (69.8)	345 (30.2)	2.21 (1.97–2.48)^a^	396 (73.2)	145 (26.8)	2.02 (1.68–2.43)^a^
Chronic back problems (ref. no.)	666 (58.3)	477 (41.7)	2.07 (1.85–2.31)^a^	375 (69.3)	166 (30.7)	1.39 (1.16–1.66)^a^
WMSDs (ref. no.)	1,118 (97.8)	25 (2.2)	1.68 (1.17–2.41)^b^	536 (99.1)	5 (0.9)	0.77 (0.32–1.84)^c^
Depression (ref. no.)	1,001 (87.6)	142 (12.4)	1.56 (1.33–1.84)^a^	472 (87.2)	69 (12.8)	1.66 (1.30–2.11)^a^
Other mental illnesses (ref. no.)	1,122 (98.2)	21 (1.8)	2.78 (1.93–4.00)^a^	528 (97.6)	13 (2.4)	3.89 (2.38–6.36)^a^
COPD (ref. no.)	1,088 (95.2)	55 (4.8)	1.64 (1.28–2.09)^a^	507 (93.7)	34 (6.3)	2.19 (1.58–3.05)^a^
Cancer (ref. no.)	1,070 (93.6)	73 (6.4)	1.36 (1.09–1.70)^a^	505 (93.3)	36 (6.7)	1.45 (1.05–2.01)^b^
Kidney failure (ref. no.)	1,091 (95.5)	52 (4.5)	1.94 (1.51–2.48)^a^	513 (94.8)	28 (5.2)	2.31 (1.62–3.31)^a^
Number of chronic diseases^a^	n	%		n	%	
0	111	10.6	1	52	10.4	1
1	212	20.2	1.57 (1.26–1.96)	108	21.6	1.73 (1.25–2.40)
2	234	22.3	2.16 (1.74–2.69)	108	21.6	2.20 (1.59–3.05)
3	207	19.7	3.00 (2.41–3.74)	87	17.4	2.87 (2.05–4.02)
4 +	286	27.2	4.44 (3.60–5.47)	144	28.9	5.22 (3.83–7.11)
**IADLs**						
SAH (ref. no.)	665 (43.5)	865 (56.5)	1.41 (1.28–1.54)^a^	677 (39.2)	1,051 (60.8)	1.62 (1.48–1.77)^a^
Diabetes (ref. no.)	1,130 (77.4)	330 (22.6)	1.48 (1.32–1.65)^a^	1,239 (74.3)	428 (25.7)	1.66 (1.51–1.83)^a^
Hypercholesterolemia (ref. no.)	1,024 (70.6)	426 (29.4)	1.26 (1.14–1.40)^a^	1,210 (74.0)	426 (26.0)	1.10 (0.99–1.21)^c^
Heart diseases (ref. no.)	1,317 (85.5)	223 (14.5)	1.75 (1.55–1.98)^a^	1,451 (83.2)	293 (16.8)	1.96 (1.76–2.18)^a^
Stroke (ref. no.)	1,452 (94.3)	88 (5.7)	1.90 (1.58–2.28)^a^	1,472 (84.4)	272 (15.6)	3.56 (3.25–3.90)^a^
Bronchial asthma (ref. no.)	1,465 (95.1)	75 (4.9)	1.21 (0.98–1.49)^c^	1,631 (93.5)	113 (6.5)	1.52 (1.29–1.79)^a^
Rheumatoid arthritis (ref. no.)	1,214 (78.8)	326 (21.2)	1.50 (1.35–1.68)^a^	1,290 (74.0)	454 (24.0)	1.83 (1.66–2.00)^a^
Chronic back problems (ref. no.)	1,055 (68.5)	485 (31.5)	1.40 (1.27–1.55)^a^	1,168 (67.0)	576 (33.0)	1.47 (1.35–1.61)^a^
WMSDs (ref. no.)	1,516 (98.4)	24 (1.6)	1.14 (0.79–1.64)^a^	1,729 (99.1)	15 (0.9)	0.68 (0.42–1.10)^c^
Depression (ref. no.)	1,366 (88.7)	174 (11.3)	1.48 (1.29–1.70)^a^	1,536 (88.1)	208 (11.9)	1.54 (1.35–1.74)^a^
Other mental illnesses (ref. no.)	1,524 (99,0)	16 (1,0)	2.14 (1.43–3.19)^a^	1,706 (97.8)	38 (2.2)	3.13 (2.52–3.88)^a^
COPD (ref. no.)	1,482 (96.2)	58 (3.8)	1.41 (1.12–1.78)^a^	1,646 (94.4)	98 (5.6)	1.89 (1.60–2.23)^a^
Cancer (ref. no.)	1,459 (94.7)	81 (5.3)	1.14 (0.93–1.40)^c^	1,637 (93.9)	107 (6.1)	1.30 (1.09–1.54)^b^
Kidney failure (ref. no.)	1,485 (96.4)	55 (3.6)	1.65 (1.31–2.09)^a^	1,667 (95.6)	77 (4.4)	1.90 (1.58–2.29)^a^
Number of chronic diseases^a^	n	%		n	%	
0	210	14.8	1	188	11.6	1
1	358	25.2	1.45 (1.23–1.70)	353	21.9	1.58 (1.34–1.87)
2	326	22.9	1.70 (1.44–1.99)	368	22.8	2.07 (1.76–2.44)
3	253	17.8	2.16 (1.83–2.56)	307	19.0	2.77 (2.35–3.28)
4 +	274	19.3	2.63 (2.23–3.09)	398	24.7	3.77 (3.22–4.41)

Categories for diseases (no vs. yes)

*SAH* Systemic arterial hypertension, heart diseases (infarction, angina, heart failure), stroke – cerebrovascular accident; chronic back problems (chronic back or neck pain, low back pain, sciatica, vertebrae or disc problems), *WMSDs* Work-related musculoskeletal disorders, *COPD* Chronic obstructive pulmonary disease, other mental illnesses (Alzheimer’s disease, dementia, schizophrenia, bipolar disorder, psychosis or obsessive–compulsive disorder), *BADLs* Basic activities of daily living, *IADLs* Instrumental activities of daily living

^d^Subjects may report more than one disease

^a^*P* < 0.001

^b^*P* < 0.05

^c^*P* > 0.05

In the crude analysis of moderate and severe IADL dependence, the dominant chronic diseases were other mental illnesses, stroke, heart disease, kidney failure, and rheumatoid arthritis (RA). These diseases were among the diseases with probabilities between 50 and 114% for the development of moderate dependence on IADLs. For severe dependence on IADLs, the chronic diseases most likely to occur for this outcome were stroke, other mental illnesses, heart disease, kidney failure, COPD, RA, diabetes, hypertension, depression, and asthma, ranging from 52 to 256% the probability of developing severe dependence on IADLs (Table 2).

The number of chronic diseases or multimorbidity maintained a linear trend toward an increase in the probability of dependence as the number of diseases increased simultaneously (Table 2).

In the adjusted analysis of each chronic disease alone (model 1), the chronic diseases associated with moderate dependence on BADLs were other mental illnesses, stroke, RA, chronic back problems, diabetes, SAH, and heart disease. In model 2, adjusted for model 1 variables (*p* < 0.05) and the number of chronic diseases and sociodemographic factors (sex, age group, education, marital status, color/race), the chronic diseases that maintained a significant association with moderate dependence on BADLs were other mental illnesses, stroke, RA, chronic back problems, and diabetes. The variable number of chronic diseases maintained the linear trend of increasing probability for the outcome as more diseases accumulated, with a 2.37 times greater possibility of occurrence of moderate dependence in BADLs for those with four or more diseases compared with the absence of chronic diseases (Table 3).

**Table 3:** Adjusted analysis of the association between chronic diseases and moderate and severe dependence in community-dwelling Brazilian older adults

**Exposure variables**	Moderate dependence		Severe dependence	
**BADLs**	Model 1PR_Adjusted_ (95% CI)	Model 2PR_Adjusted_ (95% CI)	Model 1PR_Adjusted_ (95% CI)	Model 2PR_Adjusted_ (95% CI)
SAH (ref. no.)	1.25 (1.10–1.42)	1.02 (0.85–1.21)^c^	0.97 (0.81–1.16)^c^	–
Diabetes (ref. no.)	1.40 (1.23–1.60)^a^	1.23 (1.04–1.45)^b^	1.65 (1.37–1.99)^a^	1.66 (1.28–2.16)^a^
Hypercholesterolemia (ref. no.)	0.97 (0.86–1.10)^c^	–	–	–
Heart diseases (ref. no.)	1.24 (1.06–1.45)^a^	1.04 (0.85–1.27)^c^	1.23 (0.97–1.55)^c^	–
Stroke (ref. no.)	2.21 (1.84–2.64)^a^	2.08 (1.68–2.58)^a^	6.96 (5.75–8.43)^a^	5.88 (4.50–7.69)^a^
Bronchial asthma (ref. no.)	1.11 (0.88–1.41)^c^	–	1.44 (1.01–2.04)^b^	1.59 (1.06–2.38)^b^
Rheumatoid arthritis (ref. no.)	1.67 (1.46–1.90)^a^	1.46 (1.22–1.74)^a^	1.73 (1.42–2.11)^a^	1.72 (1.29–2.28)^a^
Chronic back problems (ref. no.)	1.65 (1.45–1.87)^a^	1.46 (1.23–1.73)^a^	1.05 (0.86–1.28)^c^	–
WMSDs (ref. no.)	1.04 (0.72–1.50)^c^	–	–	–
Depression (ref. no.)	1.04 (0.88–1.24)^c^	–	1.15 (0.89–1.49)^c^	–
Other mental illnesses (ref. no.)	2.26 (1.52–3.35)^a^	2.17 (1.43–3.29)^a^	3.29 (2.07–5.24)^a^	4.11 (2.20–7.66)^a^
COPD (ref. no.)	1.11 (0.84–1.46)^c^	–	1.32 (0.90–1.93)^c^	–
Cancer (ref. no.)	1.17 (0.93–1.47)^c^	–	1.33 (0.96–1.86)^c^	–
Kidney failure (ref. no.)	1.28 (1.00–1.64)^c^	–	1.46 (1.00–2.15)^c^	–
Number of chronic diseases				
0		1		1
1		1.54 (1.16–2.05)^b^		1.63 (1.09–2.44)^b^
2		1.59 (1.16–2.18)^b^		1.37 (0.89–2.11)^c^
3		2.06 (1.44–2.95)^a^		1.78 (1.14–2.78)^b^
4 +		2.37 (1.55–3.64)^a^		1.78 (1.07–2.96)^b^
**IADLs**				
SAH (ref. no.)	1.20 (1.08–1.32)^a^	0.97 (0.83–1.12)^c^	1.25 (1.14–1.38)^a^	1.01 (0.88–1.16)^c^
Diabetes (ref. no.)	1.34 (1.19–1.50)^a^	1.27 (1.09–1.48)^b^	1.44 (1.30–1.58)^a^	1.38 (1.20–1.58)^a^
Hypercholesterolemia (ref. no.)	1.04 (0.93–1.16)^c^	–	–	–
Heart diseases (ref. no.)	1.41 (1.24–1.61)^a^	1.36 (1.15–1.61)^a^	1.38 (1.23–1.54)^a^	1.18 (1.02–1.36)^b^
Stroke (ref. no.)	1.57 (1.30–1.90)^a^	1.54 (1.21–1.95)^a^	2.90 (2.61–3.21)^a^	2.69 (2.31–3.12)^a^
Bronchial asthma (ref. no.)	–	–	1.11 (0.94–1.32)^c^	–
Rheumatoid arthritis (ref. no.)	1.27 (1.13–1.44)^a^	1.21 (1.04–1.41)^b^	1.52 (1.38–1.68)^a^	1.38 (1.20–1.59)^a^
Chronic back problems (ref. no.)	1.21 (1.08–1.35)^a^	1.12 (0.96–1.30)^c^	1.18 (1.07–1.30)^b^	1.05 (0.92–1.20)^c^
WMSDs (ref. no.)	–	–	–	–
Depression (ref. no.)	1.19 (1.03–1.38)^b^	1.40 (1.18–1.67)^a^	1.07 (0.94–1.22)^c^	–
Other mental illnesses (ref. no.)	1.76 (1.13–2.73)^b^	1.97 (1.13–3.42)^b^	2.51 (1.95–3.23)^a^	2.60 (1.76–3.82)^a^
COPD (ref. no.)	1.21 (0.96–1.52)^c^	–	1.35 (1.13–1.61)^b^	1.47 (1.20–1.80)^a^
Cancer (ref. no.)	–	–	1.18 (0.99–1.40)^c^	–
Kidney failure (ref. no.)	1.31 (1.03–1.65)^b^	1.33 (1.01–1.76)^b^	1.25 (1.04–1.51)^b^	1.53 (1.21–1.95)^a^
Number of chronic diseases				
0		1		1
1		1.39 (1.12–1.72)^b^		1.39 (1.11–1.73)^b^
2		1.35 (1.05–1.73)^b^		1.45 (1.13–1.85)^b^
3		1.62 (1.20–2.19)^b^		1.70 (1.27–2.26)^a^
4 +		1.47 (0.99–2.17)^c^		1.65 (1.16–2.36)^b^

Categories for diseases (no vs. yes)

*SAH* Systemic arterial hypertension, heart diseases (infarction, angina, heart failure), stroke – cerebrovascular accident, chronic back problems (chronic back or neck pain, low back pain, sciatica, vertebrae or disc problems), *WMSDs* Work-related musculoskeletal disorders, *COPD* Chronic obstructive pulmonary disease, other mental illnesses (Alzheimer’s disease, dementia, schizophrenia, bipolar disorder, psychosis or obsessive–compulsive disorder), *BADLs* Basic activities of daily living, *IADLs* Instrumental activities of daily living

Model 1: adjusted for all chronic diseases

Model 2: adjusted by the variables of model 1 (*p* < 0.05) + number of chronic diseases + sociodemographic factors (sex, age group, education, marital status, color/race)

^a^*P* < 0.001

^b^*P* < 0.05

^c^*P* > 0.05

For severe BADL dependence, the chronic diseases that maintained a significant association in model 1 were stroke, other mental illnesses, RA, diabetes, and bronchial asthma. In model 2, the emphasis was on stroke, other mental illnesses, RA, diabetes, and bronchial asthma. However, the variable number of chronic diseases did not follow the growth trend as the simultaneous occurrence of more chronic diseases increased, although probabilities of > 60% for the occurrence of a chronic disease and of 70% for the development of severe dependence for BADLs in the case of simultaneous illnesses of three or four and more simultaneous chronic illnesses were observed.

In model 1, the adjusted analysis for moderate dependence in IADLs followed the same trend of a higher probability of the outcome of chronic diseases for moderate dependence in BADLs: other mental illnesses and stroke. However, the chronic diseases with the highest probability had an alteration in the order of prevalence compared with the most prevalent ones for the moderate dependence for BADLs, with, for the condition in evidence, the following decreasing order of probability: heart diseases, diabetes, and kidney failure, with values > 30%. Chronic diseases, such as RA, chronic back problems, and hypertension, had probabilities between 20 and 27%. In the adjustment in model 2 for moderate dependence on IADLs, the chronic diseases that maintained a significant association and presented probabilities > 20%, being in increasing order of probability of occurrence of the outcome, were RA, diabetes, kidney failure, diseases of the heart, depression, stroke, and other mental illnesses. Regarding the number of simultaneous chronic diseases, the tendency for moderate dependence on IADLs to increase continued, with approximately 60% of the older adults who accumulated three chronic diseases likely developing moderate dependence.

Model 1, for the adjusted analysis of severe dependency in IADL, revealed significant values of the probability of occurrence of severe dependency, of 2.90 times (95CI%, 2.61–3.21) for stroke and 2.51 times (95CI%, 1.95–3.23) for other mental illnesses. The other chronic diseases maintained a significant association with severe dependence on IADLs, with probabilities of the occurrence of the outcome between 18% for chronic back problems and 52% for RA. In the adjustment of model 2, the two chronic diseases incremented in model 1 maintained a significant association for severe dependence on IADLs, that is, stroke, with 169% probability and other mental illnesses with 160% probability for the occurrence of the outcome. In addition, with the adjustment for model 2, the other chronic diseases that maintained a significant association with severe dependence for IADLs were kidney failure, COPD, diabetes, RA, and heart disease. For multimorbidity, the linear trend persisted with an increase in the probability of severe dependence for IADLs as the number of diseases increased, with a value of 70% being observed for three simultaneous chronic diseases.

The studied chronic diseases demonstrated a vital association with dependence on BADLs and IADLs. Older adults with three or more coexisting chronic diseases were 78% more likely (95% CI, 1.14–2.78) to be severely dependent on BADLs and 70% (95% CI, 1.27–2.26) more likely to be dependent on IADLs compared with their peers without chronic diseases. The same was observed for moderate dependence, and for BADLs and IADLs, the prevalences were approximately 2.06 (95% CI, 1.44–2.95) and 1.62 (95% CI, 1.20–2.19), respectively.

The results of the factor analysis are presented in Table 4. For BADLs, the KMO coefficients were 0.624 for moderate dependence and 0.632 for severe dependence. For IADL, the KMO coefficients were 0.644 for moderate dependence and 0.645 for severe dependence. Bartlett’s sphericity test showed a *p*-value < 0.001 for both, suggesting adequate factorial analysis.

**Table 4:** Factorial analysis of multimorbidity patterns in community-dwelling Brazilian older adults with moderate and severe dependence on ABVD and IADLs

	BADLs						IADLs					
Morbidities	Moderate dependence^a^			Severe dependence^b^			Moderate dependence^c^			Severe dependence^d^		
	Factor1	Factor2	Factor3	Factor1	Factor2	Factor3	Factor1	Factor2	Factor3	Factor1	Factor2	Factor3
SAH		0.53		0.64			0.61			0.61		
Diabetes		0.66		0.55			0.66			0.57		
Hypercholesterolemia		0.57		0.71			0.57			0.66		
Heart diseases		0.31		0.42					0.44	0.38		
Stroke		0.46		0.34			0.37			0.39		
Bronchial asthma			0.70			0.63			0.55			0.70
Rheumatoid arthritis	0.63				0.55			0.51			0.47	
Chronic back problems	0.67				0.59			0.53			0.62	
WMSDs	0.42				0.57			0.51			0.32	
Depression	0.53				0.50			0.61			0.60	
Other mental illnesses					0.42			0.40			0.43	
COPD			0.76			0.70			0.67			0.76
Cancer			0.30			0.30						
Kidney failure	0.33					0.44						
Variance	1.69	1.50	1.34	1.73	1.57	1.52	1.47	1.44	1.42	1.56	1.48	1.47
% variance	12.10	10.64	9.57	12.36	11.23	10.90	10.50	10.30	10.15	11.18	10.55	10,50
% accumulated	12.10	22.74	**32.31**	12.36	23.60	**34.50**	10.50	20.80	**30.95**	11.18	21.74	**32.24**

*SAH* Systemic arterial hypertension; heart diseases (infarction, angina, heart failure), stroke – cerebrovascular accident, chronic back problems (chronic back or neck pain, low back pain, sciatica, vertebrae or disc problems), *WMSDs* Work-related musculoskeletal disorders, *COPD* Chronic obstructive pulmonary disease, other mental illnesses (Alzheimer’s disease, dementia, schizophrenia, bipolar disorder, psychosis or obsessive–compulsive disorder)

Kaiser–Meyer–Olkin and Bartlett’s test: ^a^(0.624, *p* < 0.001), ^b^(0.632, *p *< 0.001), ^c^(0.644, *p* < 0.001), ^d^(0.645, *p* < 0.001)

Rotation method: Varimax with Kaiser normalization

Three factors with a multimorbidity pattern for moderate dependence were established, with an accumulated percentage of variance of 32.31%. Factor 1 included chronic diseases: chronic back problems, RA, depression, WMSDs, and kidney failure. Factor 2 included the following chronic diseases: diabetes, hypercholesterolemia, hypertension, stroke, and heart disease. Factor 3 included the following chronic diseases: COPD, bronchial asthma, and cancer.

Similarly, severe dependence on BADLs, with an accumulated percentage of the variance of 34.50%, comprised factor 1, which included the following chronic diseases: hypercholesterolemia, SAH, diabetes, heart disease, and stroke. In factor 2, chronic diseases were included: chronic back problems, WMSDs, RA, depression, and other mental illnesses. Factor 3 included the following chronic diseases: COPD, bronchial asthma, kidney failure, and cancer.

For IADLs, three factors with a multimorbidity pattern for moderate dependence were also included, with an accumulated percentage of variance of 30.95%. Factor 1 included the following chronic diseases: diabetes, SAH, hypercholesterolemia, and bronchial asthma. In factor 2, chronic diseases included depression, chronic back problems, RA, WMSDs, and other mental illnesses. In addition, factor 3 included COPD, bronchial asthma, and heart disease.

Similarly, for severe dependence on IADLs, with an accumulated percentage of variance of 32.24%, part of factor 1 included the following chronic diseases: hypercholesterolemia, SAH, diabetes, stroke, and heart disease. Factor 2 included chronic back problems, depression, RA, and other mental illnesses. Factor 3 included COPD and bronchial asthma.

## Discussion

The discrepancy in the prevalence of severity between BADL and IADL is undoubtedly related to the requirements that involve skills at different difficulty levels and the involvement of the body and cognitive structures. The former refers to physical functions, body structures and activities, and performance in individual and social contexts. In contrast, the latter involves more elaborate participation and higher concentration aspects, encompassing personal, relational and environmental aspects [31]. Functional dependence commonly follows the path of IADLs towards BADLs, corroborating our findings and confirming that the first difficulties faced by older adults occur in their routine and are related to the impairment of their cognitive function, a structuring condition of the IADL components [32].

Chronic diseases, such as other mental illnesses and stroke, are of particular relevance, as they are more disabling conditions that deserve special attention because of their potential to result in severe dependence. Both situations expose how chronic diseases can affect skills that involve cognitive capacity concerning the intentionality of performing motor activities and decision-making for autonomy and independence.

Chronic diseases significantly affect the lives of the older adults. The implications of functional decline from the simultaneous occurrence of chronic diseases may be related to the emerging concept of treatment burden, as older adults with multiple chronic diseases are more likely to use several healthcare providers simultaneously and undergo complex treatments than those without multiple chronic diseases [33]. Discriminating the association between chronic diseases and the state of dependence described in this study reveals the urgency in prioritizing primary health actions, aiming to maintain the functional capacity of this population for as long as possible, corroborating the perspective of the concept of healthy aging [34].

Mental disorders with cognitive impairment increase the risk of severe disability with little chance of recovery, further increasing the possibility of death, with a slight difference between sexes. In the presence of cognitive impairment, few disability recoveries are observed, especially in severe disabilities, regardless of the presence or absence of another chronic disease [35].

These findings corroborate the argument for improving preventive care with particular attention to the strong association between functionality and cognition and with greater attention paid to those with cognitive impairment. It is necessary to conduct extensive cohort studies to understand better the association among population aging, dementia, depression, and disability [36, 37].

The findings of this study demonstrate that cognitive impairment is the most disabling condition, confirming its importance as a primary determinant of disability. Thus, preventive care should be a priority for older adults at greater risk of loss of functionality from cognitive impairment, using recognition of physical capacity status, which may result in an opportunity for earlier intervention that would delay the onset of significant disability, preventing the progression of functional impairment [12, 35, 38].

When analyzing the condition of severe dependence for both activities studied (BADLs and IADLs), an inversion of the probabilities of higher values of the factors associated with the occurrence of the severe outcome is obtained compared with the moderate dependence; that is, the chronic disease of the stroke type appears with greater probabilities, with other mental illnesses soon after.

In this study, stroke demonstrated how impactful its occurrence was for an unfavorable outcome both for severe dependence on BADLs and IADLs, overcoming these stratum situations that, although also harmful, similar to other mental illnesses, translate into evidence of its burden in the development of severe dependence in both activities.

Globally, elevated systolic blood pressure is the single most significant risk factor for stroke (contributing 79.6 million disability-adjusted life years [DALYs] (7.7–90.8) or 55.5% (48.2–62.0) of the total DALY of stroke) [39]. Hypertension is a multifactorial chronic disease related to different outcomes, among which stroke is recognized as one of the main outcomes [40]. The Global Burden of Disease (GBD) estimates that stroke overload in older adults is expected to increase by 44% from 2004 to 2030 [41].

In 2019, 89% of the stroke burden was related to developing countries. In addition, more than 12.2 million new stroke cases are reported annually. Globally, one in four people aged > 25 years experiences a stroke in their lifetime. Over 143 million years of healthy life are lost annually due to stroke, death, and disability [42].

In developing countries, acute stroke requires more significant investment in specialized units for better management, including rehabilitation with physiotherapy, speech-language and occupational therapies, and counseling, to prevent second strokes, reduce deaths, disabilities, and the need for long-term institutional care [43].

In this study, depression was highlighted as a factor significantly associated with moderate dependence in IADLs. Assessing the degree of dependence better distinguishes the reality of the functional decline experienced by older adults, whose increase is related to higher rates of depression and decreased life satisfaction [44, 45].

In population studies, significant cognitive impairments, such as depression, dementia, and Alzheimer’s disease, contribute to disability and different states of functional dependence, emphasizing on dependence on instrumental activities [46, 47].

Gill et al. observed that in the trajectory of mental disability, it was possible to verify that among individuals with advanced stages of dementia and depression, with or without cognitive impairment, approximately 67.9% of older adults had persistent severe disability [48]. Another study demonstrated that the older adults with mental problems and cognitive deficits immediately transitioned to a disabled status and later died [49].

Older people need screening mechanisms for the discovery of mild cognitive impairment. They should be considered a “high-risk” population worthy of greater attention, given the high prevalence and the rapid progression of mild cognitive impairment to disability [2, 50].

Kidney failure was highlighted in association with moderate dependence on IADLs, emphasizing that the implications of this chronic disease for dependence are intrinsically associated with the routine activities performed by the patient, considering that kidney disease has several limitations, which may be related to complications of diabetes, other mental health problems, and stroke. This chronic disease is even more closely related to the outcome of severe dependence on IADLs, reflecting its degree of interference in activities that require more significant involvement in both motor and cognitive skills.

However, the prevalence of chronic kidney disease in Brazil remains unclear. More recent population estimates reveal approximately 1.5% of self-reported kidney diseases [51]. This statistic indicates that the adverse effects of chronic kidney disease on independence can be cumulative, causing injuries that increase a person’s suffering, especially when requiring dialysis care, imposing various restrictions and limitations on the patient, and requiring the permanent help of others [52, 53].

Hospitalization, incapacity to work, and loss of independent living are more common among patients with chronic kidney failure than in the general population [54]. Multimorbidity requires effective management beyond kidney failure itself [55, 56].

In the condition of severe dependence on BADLs, diabetes advanced as aa critical condition when the probability for the occurrence of the outcome increased, previously occupied by chronic back problems in moderate dependence. Diabetes in moderately dependent IADLs is probably also related to limitations imposed by the disease. Also, caring for older adults with diabetes requires considerable vigilance and quality monitoring, given the need to use specific medications with previous blood glucose measurement, balanced dosage, and maintenance of an adequate diet. These can provide older adults with appropriate living conditions without more significant risks of discomfort, such as hypoglycemia, which can precede falls and other complications. In contrast, uncontrolled glycemic control can evolve into systemic problems, including kidney failure and blindness, worsening functional dependence [56].

By 2040, diabetes will be the third leading cause of death in Brazil, and hyperglycemia will be the third leading cause of death. According to the GBD 2017 data for diabetes, premature deaths from diabetes considerably increased from 27.4 to 31.6%, disability due to diabetic neuropathy increased from 14.5 to 18.5%, and living with diabetes increased from 4.4 to 5.1% [57].

The detection and management of diabetes in older adults is suboptimal. The prevalence of diabetes (diagnosed and undiagnosed) increases markedly with age, from approximately 2.4% in individuals aged 20–39 years to 21.6% in individuals aged > 65 years [41].

Diabetes had the highest risk of incident disability in men (hazard ratio [HR], 3.0; 95% CI, 2.4–3.8) and women (HR, 1.7; 95% CI, 1.3–2.2). Both sexes have a considerable risk of disability [49].

In a Peruvian study, the prevalences of severe functional dependence in patients with and without diabetes were 7.1% (95% CI, 5.5%–9.0%) and 5.8 (95% CI, 5.0%–6.6%), respectively. Similarly, the prevalences of moderate functional dependence were 15.6% (95% CI, 12.7%–18.9%) and 12.4% (95% CI, 11.5%–13.3%) in patients with and without diabetes, respectively. Thus, 22.6% (95% CI, 19.4%–26.2%) of patients with diabetes had some degree of functional dependence compared with 18.1% (95% CI, 16.9%–19.4%) of patients without diabetes [58].

Chronic diseases, such as COPD, directly interfere with IADL development, as instrumental activities have several characteristics, in addition to requiring more complex ability to solve more complex situations from a cognitive point of view. COPD, in unsuccessful situations, can lead to anxiety, with respiratory repercussions and requires cardiorespiratory conditioning that is compatible with the degree of demand of the activity such as locomotion on different planes, climbing obstacles, and balance, which demands an efficient respiratory system from the older adults, capable of initiating and finishing activities, which are rarely observed in cases of COPD, even mild cases.

COPD is the third leading cause of death worldwide, causing 3.23 million deaths in 2019. Nearly 90% of COPD deaths in individuals aged < 70 years occur in low- and middle-income countries [59].

Studies have presented results that explicitly state that the greater the impairment of lung function, the more impaired the functional capacity of individuals with COPD. The greater the degree of lung capacity impairment, the greater its influence on an individual’s functional capacity [60–62].

More than three-quarters of the global COPD cases occur in low- and middle-income countries. Addressing this chronic disease is a major and significant challenge for healthcare systems in these locations. In the absence of population-wide efforts and health system reforms in these countries, where several countries have limited resources, achieving a substantial reduction in the global burden of COPD may remain difficult [63].

Brazilian estimates indicate that respiratory diseases, such as asthma and COPD, are more prevalent than other chronic respiratory diseases [64, 65], which is similar to the data from other countries [59, 66].

In this study, RA remained in the same order of importance when comparing moderate and severe dependence status for BADLs. The same did not occur for IADL outcomes.

RA is a chronic systemic autoimmune condition primarily affecting synovial joints, causing inflammation (synovitis), joint erosion, and cartilage damage. This results in a reduced functional status and disability in several patients. RA can also manifest as an extra-articular disease that affects most organs in the body, leading to higher mortality and morbidity [67].

RA is one of the main contributors to functional dependence and loss of independence in older adults, causing difficulties in maintaining their activities of daily living [68]. Data from the GBD Project identify common causes of years of healthy life lost due to disability in individuals aged > 60 years and identify osteoarthritis as one of the ten most relevant causes of disability in this population [69].

Diseases such as RA have been described as being diseases of long-term addiction, reflecting the low lethality and high disabling effect, with worse scenarios for older adults and women with diabetes as well [49, 70].

A study using measures weighted by severity found a strong and consistent relationship between accumulation of morbidities and mortality [71]. These researches indicate that the impact of morbidities on the functionality and quality of life of elderly people serve as references for monitoring public health actions, and can reflect how well cared for this population is [72]. Another study pointed out a direction of effect, that is, that multimorbidity predicts future functional decline, with physical decline being a condition for worsening multimorbidity [73]. This could perhaps explain the finding in our study of the exposure–response relationship between the number of chronic diseases and dependence on BADL and IADL.

We identified three multimorbidity patterns associated with moderate and severe dependence for the 14 chronic diseases surveyed. The factor loadings with the greatest strength of association converged, forming up to three patterns of multimorbidity that can be identified as cardio-metabolic, musculoskeletal, and respiratory, corroborating findings from a previous study based on similar data [74]. The smaller number of patterns for the respiratory component in our research is due to stratification, or the broad categories of diseases used in measurement. Factor analysis demonstrated evidence that chronic cardio-metabolic and musculoskeletal diseases tend to be more disabling, especially for dependence on IADL, both moderate and severe, and dependence on severe BADL, and for dependence on moderate BADL the highest percentage of variance (12.10%) was chronic musculoskeletal diseases, followed by cardio-metabolic diseases. The highest factor loadings converged on cardio-metabolic and musculoskeletal multimorbidity patterns, consistent with a population-based cross-sectional study carried out in Spain [75].

It is essential to mention the synergistic interaction between chronic diseases. Owing to the health emergency in the last three years worsened in the presence of chronic non-communicable diseases (CNCDs), such as moderately evidenced in the factor analysis in the present study and with the emergence of coronavirus disease 2019 (COVID-19). In one of the first published meta-analyses on this subject, Wang et al. [76] found a significant increase in the risk of worsening the new coronavirus disease among patients with multimorbidities. Studies have identified hypertension, COPD, and cardiovascular and cerebrovascular diseases as independent risk factors for COVID-19 [77].

One of the first evidence identified in the pandemic was that the magnitude and severity of cases were intensified because of other pre-existing chronic diseases. As a syndemic, COVID-19 interacts, aggravates, and is aggravated by CNCDs and existing social conditions [78].

Economically underserved populations and ethnic minority groups have higher rates of nearly all clinical risk factors that increase the severity and mortality of COVID-19 [78]. A meta-analysis study revealed the high prevalence of multimorbidities among fatal and severe cases resulting from COVID-19 [79].

Although the biological repercussions of the disease are not fully understood, respiratory, neurological, musculoskeletal, and mental health problems have affected infected individuals. Thus, health systems should expand and facilitate access to specialized outpatient and physiotherapy services and psychosocial care networks. Additionally, it is crucial to integrate these services with primary health care to guarantee integrated and longitudinal care [80].

## Limitations

The study data were almost entirely self-reported; therefore, there was a potential information bias. Nevertheless, self-reported information has been widely used and previously validated concerning studies with laboratory tests and objective examinations. Moreover, in population-based studies, it has been widely used to provide information about the severity and type of limitations experienced in different situations and contexts with a reasonable degree of reliability [31, 81–83].

## Strengths

Is the paper presented is one of the rare studies in an older adult population in a developing country that examined the particularities of the relationship between chronic diseases in isolation and multimorbidity concerning to the outcome status of the severity of functional dependence, making it possible to analyze this outcome in a more detailed and gradual manner. Thus, considering moderate and severe dependence and comparing it with older adults without dependence, it is possible to establish dependence gradients and associate them with the demands and rehabilitation actions to be offered to these vulnerable populations, which require more specific care. Because of this, the option for measuring functional dependence, characterizing it as mild, moderate, and severe, aimed to express better the functional reality of the older adults, subject providing information, and opting for a sensitive but not less specific criterion.

## Conclusion

This population-based study of Brazilian older adults aged > 60 years identifies a strong association between the outcomes of severe dependence and chronic diseases, such as dementia with or without other mental illnesses, stroke, RA, musculoskeletal diseases, diabetes, and kidney failure. When they co-occur, they seem to support evidence regarding their association with functional dependence, with greater emphasis on mental illnesses and stroke, in situations of severe disability both for IADLs and BADLs, highlighting their harmful effects.

The prevalences of moderate dependence in Brazilian older adults for BADLs are 10.2% (95% CI, 9.6–10.7) and 4.8% (95% CI, 4.4–5.2), respectively. The prevalences are 13.8% (95% CI, 13.1–14.4) and 15.6% (95% CI, 14.9%–16.2%) in moderate and severe IADL dependence, respectively.

Musculoskeletal, respiratory, and metabolic diseases are aimed at situations of moderate disability, in which worsening of dependence, in several latent cases, will probably follow the evolution of the severity status when the necessary follow-up stops.

Future studies should focus on developing interventions that incorporate a multidisciplinary approach prioritizing the recovery of functionality, which is particularly important for older adults with several chronic diseases and serious illnesses.

## Data Availability

The characterization of the National Health Survey in Brazil is published at the electronic address: https://doi.org/10.1590/1413-81232014192.14072012 The NHS questionnaire is divided into three parts: household, all residents and selected resident, and is made up of modules with topics related to health. The breakdown of topics was as follows: NHS 2013 https://www.pns.icict.fiocruz.br/questionarios/ Database Location: https://www.pns.icict.fiocruz.br/bases-de-dados/

## Abbreviations

BADLs: Basic activities of daily living
CIs: Confidence intervals
CNCDs: Chronic non-communicable diseases
COPD: Chronic obstructive pulmonary disease
COVID-19: Coronavirus disease 2019
GBD: Global Burden of Disease
HR: Hazard ratio
IADLs: Instrumental activities of daily living
KMO: Kaiser–Meyer–Olkin
NHS: National Health Survey
RA: Rheumatoid arthritis
SAH: Systemic arterial hypertension
WMSDs: Work-related musculoskeletal disorders
